# Experiments on Plant Hybrids by Gregor Mendel

**DOI:** 10.1534/genetics.116.195198

**Published:** 2016-10-07

**Authors:** Scott Abbott, Daniel J. Fairbanks

**Affiliations:** *Department of Integrated Studies, Utah Valley University, Orem, Utah 84058; † Department of Biochemistry and Molecular Biology, Monash University, Clayton, Victoria 3800, Australia

Here, translated into English, *GENETICS* republishes the original Mendel article. As discussed in the Perspectives by Daniel J. Fairbanks and Scott Abbott this translation differs from others in an attempt to be both more accurate than previous translations and also more accessible. *GENETICS* wishes to thank Scott Abbott and Daniel J. Fairbanks for their labors in presenting the scientific community with this new translation.

## Introductory Remarks

Artificial fertilisations of ornamental plants to produce new colour variants led to the experiments discussed here. The striking regularity with which the same hybrid forms reappeared whenever fertilisation took place between the same species was the stimulus for further experiments, whose objective was to follow the development of hybrids in their progeny.

Careful observers like *Kölreuter*, *Gärtner*, *Herbert*, *Lecocq*, *Wichura*, *and others* have tirelessly devoted parts of their lives to this objective. Gärtner especially, in his work “The Production of Hybrids in the Plant Kingdom,” documented very worthwhile observations, and most recently, Wichura published fundamental researches on willow hybrids. That a generally standard law for the formation and development of hybrids has not yet been successfully given is no wonder to anyone who knows the extent of the subject and who realises the difficulties with which experiments of this kind must struggle. A final determination will result only when *detailed experiments* on the most diverse plant families are available. Anyone who surveys the work in this area will be convinced that among the numerous experiments, none have been carried out in the extent and manner that would make it possible to determine the number of the various forms in which the progeny of hybrids appear, so that one could, with confidence, arrange these forms into the individual generations and determine their relative numerical relationships. Some courage is certainly required to undertake such an extensive work; nevertheless, it seems to be the only proper means to finally reach resolution of a question regarding the evolutionary history of organic forms, the importance of which must not be underestimated.

The present treatise discusses an attempt at such a detailed experiment. It was, as the task required, limited to a relatively small group of plants and was essentially completed only after the course of 8 years. Whether the plan by which the individual experiments were arranged and carried out corresponds to the given objective may be determined through a benevolent judgment.

## Selection of the Experimental Plants

The worth and validity of any experiment are determined by the suitability of the materials as well as by their effective application. In this case as well it cannot be unimportant which plant species are chosen for the experiment or the manner in which it is conducted.

The selection of the group of plants for experiments of this kind must be done with the greatest care if one does not wish to put the results in question from the beginning.

The experimental plants must necessarily

1. Possess constantly differing characters.
2. At the time of flowering, their hybrids must be protected from the action of all pollen from other individuals or be easily protected.
3. The hybrids and their progeny in the succeeding generations must not suffer any noticeable disturbance in fertility.

Adulteration through pollen from another individual, if such were to occur unrecognised in the course of the experiment, would lead to completely false conclusions. Impaired fertility or complete sterility of individual forms, like those that appear in the progeny of many hybrids, would greatly impede the experiments or thwart them completely. To recognise the relationships of the hybrid forms to one another and to their original parents, it appears to be necessary that *every* member that develops in the series in every single generation be subjected to observation.

From the beginning, special attention was given to the *Leguminosae* because of their curious floral structure. Experiments made with several members of this family led to the conclusion that the genus *Pisum* sufficiently meets the necessary requirements. Several completely independent forms of this genus possess uniform characters that are easily and certainly distinguishable, and they give rise to perfectly fertile hybrid progeny when reciprocally crossed. Disturbance by pollen from other individuals does not easily occur, as the organs of fructification are tightly enclosed by the keel and the anthers burst early in the bud so that the stigma is covered by pollen before the flower opens. This circumstance is of special importance. Other advantages that deserve mentioning are the ease of cultivating these plants in open ground and in pots, as well as their relatively short vegetative period. Artificial fertilisation is, no doubt, somewhat laborious, but it is almost always successful. For this purpose, the not yet perfectly developed flower bud is opened, the keel separated, and each stamen slowly removed with forceps, whereupon the stigma can immediately be dusted with pollen from another individual.

A total of 34 more or less different pea varieties were obtained from several seed suppliers and subjected to a 2-year trial. In one variety a few greatly distinct forms were noticed among a larger number of identical plants. The next year there was no variation among them, however, and they matched another variety obtained from the same seed supplier in every way; without doubt the seeds had been accidentally mixed. All the other varieties produced absolutely identical and constant progeny; at least in the two trial years no essential variation was noticed. From these, 22 were selected for cross-fertilisation and were cultivated annually throughout the duration of the experiments. Without exception they held true to type.

The systematic classification is difficult and uncertain. If one were to apply the strictest definition of the term species, according to which only those individuals that display precisely the same characters under precisely the same conditions belong to the same species, then no two could be counted as a single species. According to the opinion of experts in the field, however, the majority belong to the species Pisum sativum, while the others were considered and described as subspecies of P. sativum, sometimes as independent species, such as P. quadratum, P. saccharatum, and P. umbellatum. In any case, these systematic ranks are completely unimportant for the experiments described here. It is as impossible to draw a sharp line of distinction between species and varieties as it is to establish a fundamental distinction between the hybrids of species and varieties.

## Arrangement and Order of the Experiments

If two plants that are constantly different in one or more characters are united through fertilisation, the characters in common are transmitted unchanged to the hybrids and their progeny, as numerous experiments have shown; each pair of differing characters, however, unites in the hybrid to form a new character that generally is subject to variation in the progeny. To observe these variations for each pair of differing characters and to ascertain a law according to which they occur in succeeding generations was the objective of the experiment. This experiment, therefore, breaks up into just as many individual experiments as there are constantly differing characters in the experimental plants.

The different pea forms selected for fertilisation show differences in the length and colour of the stem; in the size and form of the leaves; in the placement, colour, and size of the flowers; in the length of the flower peduncles; in the colour, form, and size of the pods; in the form and size of the seeds; and in the colour of the seed coat and of the albumen. Some of these characters, however, do not permit certain and sharp separation because the difference rests on a “more or less” that is difficult to determine. Such characters could not be used for the individual experiments, which had to be limited to characters that appear clearly and decidedly in the plants. A successful result would finally show whether they all are observed as portraying identical behaviour in hybrid union and whether, as a result, a judgment is possible about those characters that typically are inferior in their importance.

The characters included in the experiments relate to:

1. The *difference in the form of the ripe seeds*. These are either spherical or somewhat rounded, and the depressions, if any, occur on the surface, and are only shallow; or they are irregularly angular and deeply wrinkled (P. quadratum).
2. The *difference in the colour of the seed albumen* (endosperm). The albumen of the ripe seeds is pale yellow, bright yellow, or orange coloured; or it possesses a more or less intensive green colour. This difference in colour is obvious to see in the seeds, since their coats are translucent.
3. The *difference in the colour of the seed coat*. This is either coloured white, a character consistently associated with white flower colour, or it is grey, grey-brown, or leather brown with or without violet spots, in which case the colour of the standard petal appears violet, that of the wings purple, and the stem at the base of the leaf axils is tinged reddish. The grey seed coats turn blackish brown in boiling water.
4. The *difference in the form of the ripe pod*. This is either simply inflated, never pinched in places, or deeply constricted between the seeds and more or less wrinkled (P. saccharatum).
5. The *difference in the colour of the unripe pod*. It is either light to dark green or coloured a bright yellow, a colour shared by stems, leaf veins, and sepals.^1^
6. The *difference in the placement of the flowers*. They are either axial, *i.e.*, distributed along the stem, or terminal, accumulated at the end of the stem in a short false umbel, in which case the upper part of the stem is more or less widened in cross-section (P. umbellatum).
7. The *difference in the length of the stem*. The length of the stem is very different in individual forms; however, for each one it is a constant character undergoing insignificant changes insofar as the plants are healthy and are raised in the same soil. In the experiments with this character, to obtain a confident difference, the long stem of 6–7 feet was united with the short one of 0.75–1.5 feet.

Each pair of the differing characters alluded to here were united through fertilisation.

For the

**Table:** 

1^st^	experiment	60	fertilisations	were	performed on	15	plants
2^nd^	“	58	“	“	“	10	“
3^rd^	“	35	“	“	“	10	“
4^th^	“	40	“	“	“	10	“
5^th^	“	23	“	“	“	5	“
6^th^	“	34	“	“	“	10	“
7^th^	“	37	“	“	“	10	“

Of a larger number of plants of the same kind, only the most vigorous were selected for fertilisation. Feeble specimens always yield uncertain results, because even in the first generation of the hybrids, and even more so in the following generations, some of the offspring either do not succeed in flowering or produce only few and inferior seeds.

Further, in all experiments reciprocal crosses were undertaken in this manner: One of the two kinds that served as seed plants for a number of fertilisations was used as the pollen plant for the other.

The plants were raised in garden beds, a small number of them in pots, and were kept in the natural upright position by means of poles, tree branches, and taut cords. For each experiment a number of potted plants were placed in a glasshouse during the flowering period. They served as a control for the main garden experiment in case of possible disturbance by insects. Among those that visit the pea plant, the beetle species Bruchus pisi could be dangerous for the experiment if it appears in large numbers. The female of this species is known to lay her eggs in the flowers and in doing so opens the keel; on the tarsi of one specimen caught in a flower, several pollen grains were obviously noticeable through a hand loupe. Here another circumstance must be noted in passing that could possibly give rise to the introduction of pollen from another individual. In rare individual cases certain parts of the otherwise completely normally developed flowers atrophy, which causes a partial exposure of the organs of fructification. Imperfect development of the keel was observed in which the style and anthers remained partially uncovered. It also sometimes happens that the pollen does not completely develop. In this case, a gradual lengthening of the style occurs during flowering until the stigma appears from the extremity of the keel. This curious phenomenon has been observed in hybrids of Phaseolus and Lathyrus.

The risk of adulteration by pollen from another individual is very slight for Pisum and can in no way disturb the result as a whole. With more than 10,000 carefully examined plants, the case of such undoubted interference occurred only a few times. Because no such disturbance was observed in the glasshouse, it may likely be supposed that Bruchus pisi and perhaps the previously alluded to abnormalities in the flower structures are to blame.

## The Form of the Hybrids

The experiments conducted with ornamental plants in past years already produced evidence that hybrids, as a rule, do not represent the precise intermediate form between the original parents. With individual characters that are particularly noticeable, like those related to the form and size of the leaves and to the pubescence of the individual parts, the intermediate form is in fact almost always apparent; in other cases, however, one of the two original parental characters possesses such an overwhelming dominance that it is difficult or quite impossible to find the other in the hybrid.

Such is exactly the behaviour of the Pisum hybrids. Each of the seven hybrid characters either resembles one of the two original parental characters so perfectly that the other one escapes observation or is so like it that a confident distinction cannot be made. This circumstance is of great importance for the determination and classification of the forms appearing among the progeny of the hybrids. In the following discussion those characters that are transmitted wholly or nearly unchanged in the hybrid association, that themselves represent the hybrid characters, are defined as *dominant*, and those that become latent in the association are defined as *recessive*. The term “recessive” was chosen because the so-named characters recede or completely disappear in the hybrid, but among the progeny thereof, as is shown later, reappear unchanged.

Further, it has been shown through all the experiments that it is completely unimportant whether the dominant character belongs to the seed plant or to the pollen plant; the hybrid form remains exactly the same in both cases. This interesting phenomenon deserves special notice, according to Gärtner, with the remark that even the most skillful expert is not able to distinguish in a hybrid which of the two united species was the seed or the pollen plant.

Of the differentiating characters introduced into the experiments, the following are dominant: (1) the spherical or somewhat rounded seed form with or without shallow indentations; (2) the yellow colour of the seed albumen; (3) the grey, grey-brown, or leather-brown colour of the seed coat, in association with violet-red flowers and reddish spotting in the leaf axils; (4) the simple inflated form of the pod; (5) the green colour of the unripe pod, associated with the same colour in the stem, leaf veins, and sepals; (6) the placement of the flowers along the stem; and (7) the length of the longer stem.

With respect to this last character, it must be remarked that the longer of the two parent stems is generally surpassed by that of the hybrid, which may be attributed to the great luxuriance that appears in all parts of the plant when stems of very different length are united in the hybrid. Thus, for example, in repeated experiments stems of 1 foot and 6 feet of length without exception produced stems in hybrid union whose length varied between 7 and 7.5 feet. *In hybrids the seed coat* is often more spotted, and the spots sometimes blend together into small bluish-violet splotches. The spotting often appears even when it is absent in the original parental characters.

The hybrid forms of the *seed shape* and *albumen* develop directly after artificial fertilisation simply through the action of the pollen from another individual. Thus they can be observed within the first experimental year, whereas all of the others appear only in the following year in the plants raised from the fertilised seeds.

## The First Generation of the Hybrids

In this generation, *along with the dominant* characters, the *recessive* characters reappear in their full individuality and do so in the determinate and pronounced average ratio of 3:1, so that of every four plants from this generation, three produce the dominant and one the recessive character. This applies without exception for all characters included in the experiment. The angular, wrinkled shape of the seeds; the green colour of the albumen; the white colour of the seed coat and of the flowers; the constriction of the pods; the yellow colour of the unripe pods and of the stems, sepals, and leaf veins; the umbel-formed inflorescence; and the dwarfed stem appear in these previously alluded to numerical relationships emerging again without any essential difference. *Transitional forms were observed in none of the experiments*.

Because the hybrids produced from reciprocal crosses acquired a wholly similar form and because no appreciable variation appeared in their further development, the results for each experiment could be combined. The ratios acquired for each pair of two differing characters are as follows:

*First experiment*: Shape of the seeds. From 253 hybrids, 7324 seeds were obtained in the second experimental year. Of these seeds 5474 were round or somewhat rounded, and 1850 were angular wrinkled. The resulting ratio is 2.96:1.
*Second experiment*: Colour of the albumen. A total of 258 plants produced 8023 seeds, 6022 yellow and 2001 green; the former relate to the latter in the ratio 3.01:1.

In these experiments one generally gets both types of seeds in each pod. For well-developed pods that on average included 6–9 seeds, it was often the case that all of the seeds were round (experiment 1) or all were yellow (experiment 2); more than 5 angular or 5 green, however, were never observed in one pod. It does not seem to make any difference if the pod develops earlier or later on the hybrid plant, if it belongs to the main stem or to a lateral one. With a few plants only single seeds developed in the pods formed first and these then had exclusively one of the two characters; in the pods that formed later, however, the ratio remained normal. As in the individual pods, the distribution of characters varied similarly among individual plants. The first 10 members from both experimental sets serve as an illustration:

**Table:** 

First experiment: shape of the seeds			Second experiment: colour of the albumen	
Plant	Round	Angular	Yellow	Green
1	45	12	25	11
2	27	8	32	7
3	24	7	14	5
4	19	10	70	27
5	32	11	24	13
6	26	6	20	6
7	88	24	32	13
8	22	10	44	9
9	28	6	50	14
10	25	7	44	18

As extremes in the distribution of the two seed characters observed in *one* plant, in the first experiment 43 seeds were round and only 2 angular, and in another 14 were round and 15 angular. In the second experiment 32 seeds were yellow and only 1 green, but also in another 20 were yellow and 19 green.

These two experiments are important for ascertaining the mean ratios because they produce especially meaningful averages with a smaller number of experimental plants. While counting the seeds, however, especially in the second experiment, some attention is required because in some seeds of several plants the green colour of the albumen is less developed and at first can be easily overlooked. The cause of the partial disappearance of the green colour has no relation to the hybrid character of the plants, as that occurs in the original parent plant as well; in addition, this peculiarity is limited only to the individual and is not inherited by the progeny. This phenomenon has often been observed in luxuriant plants. Seeds damaged by insects during their development often vary in colour and shape, but with some practice in sorting, errors are easily prevented. It is almost superfluous to mention that the pods must remain on the plant until they have ripened completely and have dried, because only then are the shape and the colour of the seeds completely developed.

*Third experiment*: Colour of the seed coat. Of 929 plants, 705 produced violet-red flowers and grey-brown seed coats; 224 had white flowers and white seed coats. This results in a ratio of 3.16:1.
*Fourth experiment*: Shape of the pods. Of 1181 plants, 882 had simply inflated and 299 had constricted pods. Hence the ratio is 2.95:1.
*Fifth experiment*: Colour of the unripe pod. The number of experimental plants was 580, of which 428 had green and 152 had yellow pods. Thus the ratio of the former to the latter is 2.82:1.
*Sixth experiment*: Position of the flowers. Of 868 cases, the flowers were located along the stem 651 times and were terminal 207 times. This ratio is 3.14:1.
*Seventh experiment*: Length of the stem. Of 1064 plants, 787 had long stems, and 277 had short ones. Hence this relative ratio is 2.84:1. In this experiment, the dwarf plants were carefully dug up and moved to separate beds. This precaution was necessary because they would have atrophied among their tall intertwining siblings. In their youngest stages they are already easily distinguished by their compact growth and their dark-green thick leaves.

If the results of all experiments are summarised, there is an average ratio between the number of forms with dominant and recessive characters of 2.98:1 or 3:1.

The dominant character can have a *double signification* here, namely that of the original parental character or that of the hybrid character. Which of these two significations occurs in each case can be determined only in the next generation. An original parental character must be transmitted unchanged to all progeny, whereas the hybrid character must follow the same behaviour as observed in the first generation.

## The Second Generation of the Hybrids

Those forms that preserve the recessive character in the first generation do not vary in the second generation in relation to that character; they remain *constant* in their progeny.

This is not the case for those that possess the dominant character in the first generation. Of these *two*-*thirds* yield progeny that carry the dominant and recessive character in the ratio 3:1 and thus show the same behaviour as the hybrid forms; only *one*-*third* remains constant with the dominant character.

The individual experiments produced the following results:

*First experiment*: Of 565 plants raised from round seeds of the first generation, 193 produced only round seeds and thus remained constant in this character; 372, however, simultaneously produced round and angular seeds in the ratio 3:1. Thus the number of hybrid types relative to the number of constant types is 1.93:1.
*Second experiment*: Of 519 plants raised from seeds whose albumen in the first generation had the yellow colour, 166 produced exclusively yellow; 353, however, produced yellow and green seeds in the ratio 3:1. This resulted in division of hybrid and constant forms in the ratio 2.13:1.

For each of the following experiments, 100 plants were selected that retained the dominant character in the first generation, and to test its signification, 10 seeds from each were cultivated.

*Third experiment*: The progeny of 36 plants produced exclusively grey-brown seed coats; from 64 plants some with grey-brown and some with white seed coats were produced.
*Fourth experiment*: The progeny of 29 plants had only simply inflated pods; of 71, however, some had inflated and some had constricted pods.
*Fifth experiment*: The progeny of 40 plants had only green pods; from those of 60 plants some had green and some had yellow pods.
*Sixth experiment*: The progeny of 33 plants had flowers located only along the stem; of 67, however, some had flowers located along the stem, and some had terminal flowers.
*Seventh experiment*. The progeny of 28 plants produced long stems; from 72 plants some had long stems and some had short stems.

In each of these experiments a particular number of plants with the dominant character is constant. For determination of the ratio in which segregation takes place for the forms with the constantly permanent character, the first two experiments are of special importance because a larger number of plants could be compared. The ratios 1.93:1 and 2.13:1, taken together, result almost precisely in the average ratio 2:1. The sixth experiment has almost an identical result; in the others the ratio fluctuates more or less, as must be expected given the small number of 100 experimental plants. The fifth experiment, which showed the largest deviation, was repeated and then, instead of the ratio 60:40, produced the ratio 65:35. *The average ratio 2:1 consequently appears certain*. Thus it is proved that of each form possessing the dominant character in the first generation, two-thirds carry the hybrid character; one-third, however, remains constant with the dominant character.

The ratio 3:1, which results in the distribution of the dominant and recessive characters in the first generation, resolves *then for all experiments into the ratio 2:1:1*, if one simultaneously distinguishes the dominant character in its signification as a hybrid character and as an original parental character. Because the members of the first generation arise directly from the seeds of the hybrids, *it now becomes apparent that the hybrids from each pair of differing characters form seeds*, *of which one-half again develops the hybrid form*, *whereas the other yields plants that remain constant and produce in equal parts the dominant and the recessive character*.

## The Subsequent Generations of the Hybrids

The ratios according to which the offspring of the hybrids develop and segregate in the first and second generations are valid, in all probability, for all subsequent generations. The first and second experiments have now been continued through six generations; the third and seventh through five generations; and the fourth, fifth, and sixth through four generations, although beginning from the third generation with a smaller number of plants, without any noticeable deviation. The progeny of the hybrids in each generation segregated into hybrid and constant forms according to the ratio 2:1:1.

If ***A*** represents one of the two constant characters, for example the dominant, ***a*** the recessive, and ***Aa*** the hybrid form in which the two are united, then the expression

A + 2Aa + a

shows the developmental series for the progeny of the hybrids of each pair of divergent characters.

The observations made by Gärtner, Kölreuter, and others that hybrids possess the tendency to revert to the original parent species are confirmed by the experiments herein discussed. It can be shown that the number of hybrids descended from fertilisation significantly decreases from generation to generation, without completely disappearing, however, when compared to the number of forms and their progeny that have become constant. If one assumes that on average all plants in all generations have equally high fertility, and if one considers further that every hybrid forms seeds of which half arise again as hybrids, whereas the other half becomes constant with both characters in equal parts, then the numeric ratios for the progeny in each generation can be shown by the following tabulation, where ***A*** and ***a*** again represent the two original characters and ***Aa*** represents the hybrid form. For the sake of brevity, assume that every plant in every generation forms only four seeds.

**Table:** 

Generation	***A***	***Aa***	***a***	Given as Ratio
				***A***:***Aa***:***a***
1	1	2	1	1:2:1
2	6	4	6	3:2:3
3	28	8	28	7:2:7
4	120	16	120	15:2:15
5	496	32	496	31:2:31
*n*				2*^n^* − 1:2:2*^n^* − 1

In the 10th generation, for example, there are 2*^n^* – 1 = 1023. Of every 2048 plants that arise from this generation, there are 1023 that are constant for the dominant character, 1023 with the recessive character, and only two hybrids.

## The Progeny of the Hybrids in Which Several Differing Characters are Combined

For the experiments just discussed, plants were used that differed in only one essential character. The next objective consisted of researching whether the developmental law found for each pair of differing characters was valid when several different characters are united in the hybrid through fertilisation.

As for the form of the hybrids in this case, the experiments agreed in showing that the hybrid more closely resembles the original parent plant that possesses the larger number of dominant characters. If, for example, the seed plant has a short stem, terminal white flowers, and simple inflated pods whereas the pollen plant has a long stem, violet-red flowers along the stem, and constricted pods, then the hybrid reflects the seed plant only in the form of the pod; in the other characters it is identical to the pollen plant. If one of the two original parents possesses only dominant characters, then the hybrid is hardly or not at all distinguishable from it.

Two experiments were carried out with a larger number of plants. In the first experiment the original parent plants differed in the shape of the seeds and in the colour of the albumen, and in the second experiment they differed in the shape of the seeds, the colour of the albumen, and the colour of the seed coat. Experiments with seed characters lead to the simplest and most certain results.

To give an easier overview of these experiments, the differing characters of the seed plant are designated with ***A***, ***B***, and ***C***; those of the pollen plant with ***a***, ***b***, and ***c***; and the hybrid forms of these characters with ***Aa***, ***Bb***, and ***Cc***:

**Table:** 

First experiment	
***AB*** seed plant	***ab*** pollen plant
***A*** round shape	***a*** angular shape
***B*** yellow albumen	***b*** green albumen

The seeds derived from fertilisation appeared round and yellow, resembling those of the seed plant. The plants raised from them produced seeds of four kinds that were often together in one pod. In total 556 seeds were produced from 15 plants; of these there were 315 round and yellow, 101 angular and yellow, 108 round and green, and 32 angular and green.

All of them were cultivated the next year. Of the round yellow seeds 11 did not produce plants and 3 plants did not produce seeds. Among the remaining plants, there were 38 round yellow seeds, ***AB***; 65 round yellow and green seeds, ***ABb***; 60 round yellow and angular yellow seeds, ***AaBb***; and 138 round yellow and green, angular yellow and green seeds, ***AaBb***.

From the angular yellow seeds 96 plants produced seeds, of which 28 had only angular yellow seeds, ***aB***; and 68 had angular, yellow and green seeds, ***aBb***.

From 108 round green seeds, 102 produced fruiting plants, from which there were 35 with only round green seeds, ***Ab***; and 67 with round and angular green seeds, ***Aab***.

The angular green seeds produced 30 plants with exactly this same type of seeds; they remained constant, ***ab***.

The progeny of the hybrids thus appeared in nine different forms and some in greatly unequal numbers. The following is the result when these are grouped and arranged:

**Table:** 

38	plants	designated as	***AB***
35	“	“	***Ab***
28	“	“	***aB***
30	“	“	***ab***
65	“	“	***ABb***
68	“	“	***aBb***
60	“	“	***AaB***
67	“	“	***Aab***
138	“	“	***AaBb***

All of the forms can be brought into three essentially different divisions. The first one includes those with the designations ***AB***, ***Ab***, ***aB***, ***ab***; they possess only constant characters and vary no more in subsequent generations. Each of these forms is represented, on average, 33 times. The second group includes the forms ***ABb***, ***aBb***, ***AaB***, ***Aab***; these are constant in one character, hybrid in the other, and vary in the next generation only for the hybrid character. Each of them appears on average 65 times. The ***AaBb*** form appears 138 times, is hybrid for both characters, and behaves exactly like the hybrid from which it is derived.

If one compares the number of forms that occur in each of these divisions, the average ratios 1:2:4 are unmistakable. The numbers 33, 65, 138 are very close approximations of the ratio numbers 33, 66, 132.

The developmental series thus consists of nine classes. Four of them appear only one time each and are constant for both characters; the forms ***AB***, ***ab*** resemble the original parents; the other two represent the remaining possible constant combinations between the unions of characters ***A***, ***a***, ***B***, ***b***. Four classes appear twice each and are constant for one character and hybrid for the other. One class occurs four times and is hybrid for both characters. Thus the progeny of the hybrids, when two pairs of differing characters are combined in them, develop according to these terms:

AB + Ab + aB + ab + 2ABb + 2aBb + 2AaB + 2Aab + 4AaBb.

This developmental series is indisputably a combination series in which the two developmental series for the characters ***A*** and ***a***, ***B***, and ***b*** are associated term by term. The total number of classes in the series is produced through combining the terms:

$$\begin{array}{l} A+2\mathrm{Aa}+a\\ B+2\mathrm{Bb}+b. \end{array}$$

**Table:** 

Second experiment	
***ABC*** seed plant	***abc*** pollen plant
***A*** round form	***a*** angular form
***B*** yellow albumen	***b*** green albumen
***C*** grey-brown seed coat	***c*** white seed coat

This experiment was conducted in a manner quite similar to the previous one. Of all the experiments it required the most time and effort. A total of 687 seeds were produced from 24 hybrids, all of which were spotted, coloured grey-brown or grey-green, and round or angular. Of those, 639 plants produced seeds the following year and, as further researches showed, among them there were the following:

**Table:** 

8	plants	***ABC***	22	plants	***ABCc***	45	plants	***ABbCc***
14	“	***ABc***	17	“	***AbCc***	36	“	***aBbCc***
9	“	***AbC***	25	“	***aBCc***	38	“	***AaBCc***
11	“	***Abc***	20	“	***abCc***	40	“	***AabCc***
8	“	***aBC***	15	“	***ABbC***	49	“	***AaBbC***
10	“	***aBc***	18	“	***ABbc***	48	“	***AaBbc***
10	“	***abC***	19	“	***aBbC***			
7	“	***abc***	24	“	***aBbc***			
			14	“	***AaBC***	78	“	***AaBbCc***
			18	“	***AaBc***			
			20	“	***AabC***			
			16	“	***Aabc***			

The developmental series includes 27 classes. Of those 8 are constant for all characters and each appears on average 10 times; 12 are constant for two characters, hybrid for the third, each appearing on average 19 times; 6 are constant for one character, hybrid for the other two, each of them occurring on average 43 times; and one form appears 78 times and is hybrid for all characters. The ratio 10:19:43:78 appears so near to the ratio 10:20:40:80 or 1:2:4:8 that the latter without doubt represents the true values.

The development of the hybrids, when their original parents are different in three characters, results thus according to the terms

$$\begin{array}{l} \mathrm{ABC}\;+\;\mathrm{ABc}\;+\;\mathrm{AbC}\;+\;\mathrm{Abc}\;+\;\mathrm{aBC}\;+\;\mathrm{aBc}\;+\;\mathrm{abC}\;+\;\mathrm{abc}\;+\;2\mathrm{ABCc}+\;2\mathrm{AbCc}\;+\;2\mathrm{aBCc}\;+\;2\mathrm{abCc}\;+\;2\mathrm{ABbC}\;+\;2\mathrm{ABbc}\;+\;2\mathrm{aBbC}+\;2\mathrm{aBbc}+2\mathrm{AaBC}\;+\;2\mathrm{AaBc}\;+\;2\mathrm{AabC}\;+\;2\mathrm{Aabc}\;+\;4\mathrm{ABbCc}+\;4\mathrm{aBbCc}+4\mathrm{AaBCc}\;+\;4\mathrm{AabCc}\;+\;4\mathrm{AaBbC}\;+\;4\mathrm{AaBbc}+\;8\mathrm{AaBbCc}. \end{array}$$

Here too is a combination series in which the developmental series for the characters ***A*** and ***a***, ***B*** and ***b***, ***C*** and ***c*** are associated with each other. The terms

$$\begin{array}{l} A\;+\;2\mathrm{Aa}\;+\;a\\ B\;+\;2\mathrm{Bb}\;+\;b\\ C\;+\;2\mathrm{Cc}\;+\;c \end{array}$$

reflect all classes in the series. The constant combinations that occur therein correspond to all combinations that are possible between the characters ***A***, ***B***, ***C***, ***a***, ***b***, ***c***; two of them, ***ABC*** and ***abc*,** resemble the two original parental plants.

In addition, various other experiments were undertaken with a smaller number of experimental plants in which the rest of the characters were associated in twos and threes in the hybrids; all produced approximately the same results. There is, then, no doubt that for all of the characters admitted into the experiments the following sentence is valid: *The progeny of hybrids in which several essentially differing characters are united represent the terms of a combination series in which the developmental series for each pair of differing characters are combined*. Simultaneously it thus is shown that *the behaviour of each pair of differing characters in hybrid association is independent of other differences between the two original parental plants*.

If *n* represents the number of the characteristic differences in the two original parent plants, then 3*^n^* yields the number of classes in the combination series, 4*^n^* the number of individuals that belong to the series, and 2*^n^* the number of combinations that remain constant. Thus, for example, if the original parents have four different characters, the series includes 3^4^ = 81 classes, 4^4^ = 256 individuals, and 2^4^ = 16 constant forms; or in other words, among 256 progeny from hybrids there are 81 different combinations, of which 16 are constant.

All constant combinations that are possible in Pisum through combining the seven characters previously alluded to were actually produced through repeated crosses. Their number is given as 2^7^ = 128. Simultaneously, factual evidence is produced *that constant characters occurring in different forms of a plant genus can*, *through repeated artificial fertilisation*, *occur in all possible combinations according to the rules of combination*.

Experiments on the flowering time of the hybrids are not yet concluded. It is already possible to note, in that regard, that flowering takes place at a time almost exactly intermediate between that of the seed and the pollen plant, and the development of the hybrids in relation to this character will probably follow in the same manner as for the other characters. The forms chosen for experiments of this kind must differ in the mean flowering time by at least 20 days; further, it is necessary that the seeds when cultivated are placed at the same depth in the earth to produce simultaneous germination; and further, large fluctuations in temperature during the entire flowering time must be taken into account to explain the resulting partial acceleration or retardation of flowering. Obviously this experiment has several difficulties that must be overcome, requiring great attention.

If we endeavour to summarise the results, we find that for those differing characters that admit easy and certain differentiation of the experimental plants, *we observe completely identical behaviour* in hybrid union. One-half of the progeny of the hybrids for each pair of differing characters is also hybrid, whereas the other half is constant in equal proportions for the characters of the seed and pollen plants. If several differing characters are united in one hybrid through fertilisation, the progeny constitute the members of a combination series in which the developmental series for all pairs of differing characters are united.

The perfect identity shown by all characters tested in the experiment fully permits and justifies the assumption that the same behaviour applies to other characters that appear less sharply in the plants and thus could not be included in the individual experiments. An experiment with flower peduncles of differing lengths on the whole produced a rather satisfactory result, although distinction and classification of the forms could not be effected with the same certainty that is indispensable for correct experiments.

## The Fertilising Cells of the Hybrids

The results of the initial experiments led to further experiments whose success appeared capable of throwing light on the nature of the germ and pollen cells of the hybrids. An important point of reference is offered in Pisum by the circumstance that constant forms appear in the progeny of its hybrids and because they do so in all combinations of the united characters. Through experience, we find it to be invariably confirmed that constant progeny can be formed only when the germ cells and the fertilising pollen are the same, in that both are equipped with the ability to create perfectly equal individuals, as is the case with normal fertilisation of pure species. We must then treat it as necessary that the very same factors combine in the production of constant forms in the hybrid plant. Because the different constant forms are produced in *one* plant, even in *one* flower of the plant, it appears logical to assume that in the ovaries of the hybrids as many germ cells (germinal vesicles) and in the anthers as many pollen cells form as there are possible *constant* combination forms and that these germ and pollen cells correspond to the individual forms in their internal nature.

In fact, it can be shown theoretically that this assumption would be thoroughly ample to account for the development of the hybrids in individual generations, if one were simultaneously allowed to assume that the different kinds of germ and pollen cells are, on average, formed in equal numbers in the hybrid.

To test these assumptions experimentally, the following experiments were chosen: Two forms that differed constantly in the shape of the seeds and in the colour of the albumen were united through fertilisation. If the differing characters are once again represented as ***A***, ***B***, ***a***, ***b***, one has

**Table:** 

***AB*** seed plant	***ab*** pollen plant
***A*** round shape	***a*** angular shape
***B*** yellow albumen	***b*** green albumen

The artificially fertilised seeds were cultivated along with seeds of the two original parent plants, and the most vigorous specimens were selected for reciprocal crosses. The fertilisations were (1) the hybrid with the pollen of ***AB***, (2) the hybrid with the pollen of ***ab***, (3) ***AB*** with the pollen of the hybrid, and (4) ***ab*** with the pollen of the hybrid.

For each of these four experiments, all the flowers of three plants were fertilised. If the above assumption is true, then the germ and pollen cells must develop as forms ***AB***, ***Ab***, ***aB***, ***ab*** in the hybrid and be united as (1) the germ cells ***AB***, ***Ab***, ***aB***, ***ab*** with the pollen cell ***AB***; (2) the germ cells ***AB***, ***Ab***, ***aB***, ***ab*** with the pollen cell ***ab***; (3) the germ cell ***AB*** with the pollen cells ***AB***, ***Ab***, ***aB***, ***ab***; and (4) the germ cell ***ab*** with the pollen cells ***AB***, ***Ab***, ***aB***, ***ab***.

From each of these experiments, then, only the following forms could emerge: (1) ***AB***, ***ABb***, ***AaB***, ***AaBb***; (2) ***AaBb***, ***Aab***, ***aBb***, ***ab***; (3) ***AB***, ***ABb***, ***AaB***, ***AaBb***; and (4) ***AaBb***, ***Aab***, ***aBb***, ***ab***.

Further, if the individual forms of the germ and pollen cells of the hybrid were formed on average in equal numbers, then in each experiment the four previously stated combinations necessarily would be equal in their numerical relationships. A perfect accordance of the numerical ratios was not expected, however, because in every fertilisation, normal ones included, individual germ cells remain undeveloped or later atrophy, and even some of the well-developed seeds do not succeed in germinating after cultivation. Also, this assumption is limited in that the formation of the different germ and pollen cells merely approaches equality in numbers and not that every individual hybrid reaches such numbers with mathematical precision.

The *first and second* experiments had the main purpose of verifying the nature of the hybrid germ cells and the *third and fourth* experiments of determining that of the pollen cells. As the above tabulation shows, the first and third experiments, and the second and fourth as well, should produce quite the same combinations; and, to some extent, these results should be partially apparent as early as the second year in the shape and colour of the artificially fertilised seeds. In the first and third experiments, the dominant characters of shape and colour, ***A*** and ***B***, appear in each combination, one part in constant association and the other part in hybrid union with the recessive characters ***a*** and ***b***, and because of this they must impress their characteristic upon all of the seeds. All the seeds must therefore, if this assumption is true, appear round and yellow. In the second and fourth experiments, however, one combination is hybrid in shape and colour and the seeds are round and yellow; another is hybrid in shape and constant in the recessive character of colour and the seeds are round and green; the third is constant in the recessive character of shape and hybrid in colour and the seeds are angular and yellow; and the fourth is constant in both recessive characters and the seeds are angular and green. In these two experiments, therefore, four seed types were expected, namely round yellow, round green, angular yellow, and angular green.

The yield corresponds to these requirements perfectly.

There were obtained in the first experiment 98 exclusively round yellow seeds; in the third experiment, 94 exclusively round yellow seeds; in the second experiment, 31 round yellow, 26 round green, 27 angular yellow, and 26 angular green seeds; and in the fourth experiment, 24 round yellow, 25 round green, 22 angular yellow, and 27 angular green seeds.

There was no longer any doubt about a favourable result, but the next generation would produce the final determination. Of the cultivated seeds, in the following year, 90 plants in the first experiment and 87 in the third experiment formed seeds. Of these, there were in the

**Table:** 

First Experiment	Third Experiment		
20	25	round yellow seeds. . . . . . . . . . . . . . . . . . .	***AB***
23	19	round yellow and green seeds. . . . . . . . . . .	***ABb***
25	22	round and angular, yellow seeds. . . . . . . . . .	***AaB***
22	21	round and angular, yellow and green seeds. .	***AaBb***

In the second and fourth experiments the round and yellow seeds produced plants with round and angular, yellow and green seeds, ***AaBb***. From the round green seeds, plants were produced with round and angular green seeds, ***Aab***. The angular yellow seeds produced plants with angular yellow and green seeds, ***aBb***. From the angular green seeds, plants were raised that produced once again only angular green seeds, ***ab***.

Although some seeds likewise did not germinate in these two experiments, the numbers found in the previous year could not be changed by that as each kind of seed produced plants that, in relation to their seeds, were similar among themselves and different from the others. Therefore, there were produced from the

**Table:** 

Second Experiment	Fourth Experiment						
31	24	plants	with	seeds	of the	form	***AaBb***
26	25	“	“	“	“	“	***Aab***
27	22	“	“	“	“	“	***aBb***
26	27	“	“	“	“	“	***aB***

In all of the experiments, then, all forms appeared as this assumption required and, in fact, in nearly the same numbers.

In another trial the characters of *flower colour and stem length* were admitted into the experiments and the selection designed so that in the third experimental year each character would appear in *half* of all plants if the above assumption were true. ***A***, ***B***, ***a***, ***b*** serve once again as designations for the different characters: ***A***, flowers violet-red; ***a***, flowers white; ***B***, stem long; and ***b***, stem short.

The form ***Ab*** was fertilised with ***ab***, producing the hybrid ***Aab***. Further, ***aB*** was also fertilised with ***ab***, producing the hybrid ***aBb***. In the second year the hybrid ***Aab*** was used as the seed plant for further fertilisation and the other hybrid ***aBb*** as the pollen plant: seed plant, ***Aab***; pollen plant, ***aBb***; possible germ cells, ***Ab***, ***ab***; and pollen cells, ***aB***,***ab***.

From the fertilisation between the possible germ and pollen cells, four combinations should appear, namely

AaBb + aBb + Aab + ab.

It thus becomes evident that according to the above assumption, in the third experimental year, of all plants, half should have violet-red flowers (***Aa***), groups 1 and 3; half should have white flowers (***a***), groups 2 and 4; half should have a long stem (***Bb***), groups 1 and 2; and half should have a short stem (***b***), groups 3 and 4.

From 45 fertilisations in the second year, 187 seeds were produced, from which 166 plants succeeded in flowering in the third year. Of those, the individual groups appeared in the following numbers:

**Table:** 

Group	Flower colour	Stem	No. of times
1	Violet-red	Long	47
2	White	Long	40
3	Violet-red	Short	38
4	White	Short	41

Thus the violet-red flower colour (***Aa***) appeared in 85 plants, the white flower colour (***a***) appeared in 81 plants, the long stem (***Bb***) appeared in 87 plants, and the short stem (***b***) appeared in 79 plants.

The proposed theory finds ample confirmation in this experiment as well.

For the characters of the *pod form*, *pod colour*, *and flower placement*, smaller experiments were made and completely concurring results were produced. All combinations possible through the union of different characters appeared as expected and in nearly equal numbers.

Thus through experimental means the assumption is justified *that pea hybrids form germ and pollen cells that, according to their nature, correspond in equal numbers to all the constant forms that arise from the combination of characters united through fertilisation.*

The different forms among the progeny of the hybrids, as well as the numerical ratios in which they are observed, find a sufficient explanation in the principle just shown. The simplest case is offered by the developmental series for *each pair of differing characters*. It is known that this series is defined by the expression ***A*** + **2*Aa*** + ***a***, in which ***A*** and ***a*** signify the forms with constant differing characters and ***Aa*** signifies the hybrid form of both. It includes four individuals among the three different classes. In their formation, pollen and germ cells of the forms ***A*** and ***a*** occur in equal proportions on average in fertilisation, and thus each form appears twice, since four individuals are formed. Therefore, participating in fertilisation are the pollen cells, ***A*** + ***A*** + ***a*** + ***a***; and the germ cells, ***A*** + ***A*** + ***a*** + ***a***.

It is a matter of chance which of the two kinds of pollen unites with each individual germ cell. According to the rules of probability, in the average of many cases, each pollen form ***A*** and ***a*** unites equally often with a germ cell form ***A*** and ***a***; thus one of the two pollen cells ***A*** will converge with a germ cell ***A*** during the fertilisation and the other with a germ cell ***a***, and, in the same way, one pollen cell ***a*** will unite with a germ cell ***A*** and the other with ***a***:

Pollen cells


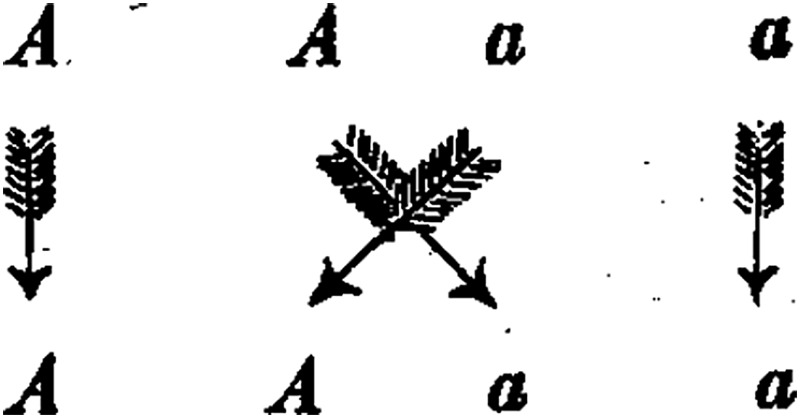


Germ cells

The result of fertilisations can be clearly illustrated if the designations for united germ and pollen cells are shown as fractions, with the pollen cells above the line, the germ cells below. Thus, in this case

$$\frac{A}{A}+\frac{A}{a}+\frac{a}{A}+\frac{a}{a}.$$

In the first and fourth classes the germ and pollen cells are the same, so the products of their association must be constant, ***A*** and ***a***. With the second and third classes, however, once again a union of the two differing original parental characters takes place, and hence the forms that appear from this fertilisation are completely identical to the hybrid from which they are derived. *Consequently*, *a repeated hybridisation takes place*. This accounts for the striking phenomenon that the hybrids are able, like the two original parental forms, to produce progeny that are identical to themselves; ***A/a*** and ***a/A*** both produce the same combination ***Aa***, because, as alluded to earlier, it makes no difference for the result of fertilisation which of the two characters belongs to the pollen or germ cells. Thus,

$$\frac{A}{A}+\frac{A}{a}+\frac{a}{A}+\frac{a}{a}=A\;+\;2\mathrm{Aa}\;+\;a.$$

This is the *average* course for the self-fertilisation of hybrids when two differing characters are united in them. In individual flowers and in individual plants, the condition through which members of the series are formed, however, can undergo alterations that are not insignificant. Except for the fact that the numbers of both types of germ cells in the ovary can be supposed only on average to occur equally, it remains wholly left to chance which of the two kinds of pollen fertilises each individual germ cell. Thus the individual values necessarily undergo fluctuations and even extreme cases are possible as alluded to earlier in the experiments on seed shape and the colour of the albumen. The true numerical ratios can be derived only as the mean from the sum of the largest possible number of individual values; the larger their number is, the more mere chance effects are eliminated.

The development series for hybrids in which *two kinds of differing characters* are associated includes nine different forms with 16 individuals, namely ***AB*** + ***Ab*** + ***aB*** + ***ab*** + **2*ABb*** + **2*aBb*** + **2*AaB*** + **2*Aab*** + **4*AaBb***. Between the different characters of the original parent plants ***A***, ***a*** and ***B***, ***b***, four constant combinations are possible, and thus the hybrid produces the corresponding four forms of germ and pollen cells, ***AB***, ***Ab***, ***aB***, ***ab***, and each of them will, on average, come into fertilisation four times, since 16 individuals are produced in the series. Thus, taking part in fertilisation are the pollen cells, ***AB*** + ***AB*** + ***AB*** + ***AB*** + ***Ab*** + ***Ab*** + ***Ab*** + ***Ab*** + ***aB*** + ***aB*** + ***aB*** + ***aB*** + ***ab*** + ***ab*** + ***ab*** + ***ab***; and the germ cells, ***AB*** + ***AB*** + ***AB*** + ***AB*** + ***Ab*** + ***Ab*** + ***Ab*** + ***Ab*** + ***aB*** + ***aB*** + ***aB*** + ***aB*** + ***ab*** + ***ab*** + ***ab*** + ***ab***.

In the average course of fertilisation, each pollen form unites equally often with every germ cell form, and thus each of the four pollen cells ***AB*** unites once with each of the germ cell types ***AB***, ***Ab***, ***aB***, ***ab***. In precisely the same way, the union of the other pollen cells of forms ***Ab***, ***aB***, ***ab*** with all the other germ cells takes place. Consequently, one obtains

$$\frac{\mathrm{AB}}{\mathrm{AB}}+\frac{\mathrm{AB}}{\mathrm{Ab}}+\frac{\mathrm{AB}}{\mathrm{aB}}+\frac{\mathrm{AB}}{\mathrm{ab}}+\frac{\mathrm{Ab}}{\mathrm{AB}}+\frac{\mathrm{Ab}}{\mathrm{Ab}}+\frac{\mathrm{Ab}}{\mathrm{aB}}+\frac{\mathrm{Ab}}{\mathrm{ab}}+\frac{\mathrm{aB}}{\mathrm{AB}}+\frac{\mathrm{aB}}{\mathrm{Ab}}+\frac{\mathrm{aB}}{\mathrm{aB}}+\frac{\mathrm{aB}}{\mathrm{ab}}+\frac{\mathrm{ab}}{\mathrm{AB}}+\frac{\mathrm{ab}}{\mathrm{Ab}}+\frac{\mathrm{ab}}{\mathrm{aB}}+\frac{\mathrm{ab}}{\mathrm{ab}},$$

or

$$\begin{array}{l} \mathrm{AB}+\mathrm{ABb}+\mathrm{AaB}+\mathrm{AaBb}+\mathrm{ABb}+\mathrm{Ab}+\mathrm{AaBb}+\mathrm{Aab}+\;\mathrm{AaB}+\mathrm{AaBb}+\mathrm{aB}+\mathrm{aBb}+\mathrm{AaBb}+\mathrm{Aab}+\mathrm{aBb}+\mathrm{ab}\\ =\mathrm{AB}+\mathrm{Ab}+\mathrm{aB}+\mathrm{ab}+2\mathrm{ABb}+2\mathrm{aBb}+2\mathrm{AaB}+2\mathrm{Aab}+\;4\mathrm{AaBb}. \end{array}$$

The developmental series for hybrids can be accounted for in a quite similar manner when *three kinds of differing characters* are combined. The hybrid forms eight different types of germ and pollen cells ***ABC***, ***ABc***, ***AbC***, ***Abc***, ***aBC***, ***aBc***, ***abC***, ***abc*** and once again each pollen type unites on average once with each germ cell type.

The law of combination of the differing characters, by which the development of hybrids results, finds its *foundation and explanation* accordingly in the conclusive principle that hybrids produce germ and pollen cells corresponding in equal number to all constant forms that arise from the combination of the characters united through fertilisation.

## Experiment on Hybrids of Other Plant Species

The objective for further experiments will be to ascertain whether the developmental law found for Pisum is also valid for hybrids of other plants. For this purpose several experiments were recently initiated. Two smaller experiments with species of Phaseolus have been concluded and they deserve mentioning here.

One experiment with Phaseolus vulgaris and Ph. nanus L. produced a completely corresponding result. Ph. nanus had, along with a dwarf stem, green, simply inflated pods; Ph. vulgaris, however, had a 10- to 12-foot long stem and yellow-coloured pods that were constricted at the time of ripening. The numerical ratios in which the different forms occurred in the individual generations were the same as in Pisum. The development of constant combinations also resulted according to the law of simple combination of characters, precisely as is the case in *Pisum*. The results were as follows:

**Table:** 

Constant combination	Stem	Colour of the unripe pod	Form of the ripe pod
1	Long	Green	Inflated
2	Long	Green	Constricted
3	Long	Yellow	Inflated
4	Long	Yellow	Constricted
5	Short	Green	Inflated
6	Short	Green	Constricted
7	Short	Yellow	Inflated
8	Short	Yellow	Constricted

Green pod colour, inflated form of the pod, and the long stem were dominant characters, as in Pisum.

Another experiment with two very different Phaseolus species had only partial success. Serving as the *seed plant* was Ph. nanus L., a very constant species with white flowers on short racemes and small white seeds in straight, inflated, and smooth pods; and serving as the *pollen plant* was Ph. multiflorus W. with a tall coiling stem, purple-red flowers on very long racemes, rough sickle-shaped bent pods, and large seeds that are spotted and mottled black on a peach-flower-red background.

The hybrid was most similar to the pollen plant; only the flowers appeared less intensively coloured. Its fertility was very limited: Of 17 plants that together produced many hundreds of flowers, a total of only 49 seeds were harvested. These were of intermediate size and retained markings resembling Ph. multiflorus; the background colour too was not essentially different. In the next year 44 plants were produced, of which only 31 succeeded in flowering. All characters of Ph. nanus that were latent in the hybrid reappeared in different combinations, but their ratios fluctuated greatly because of the small number of experimental plants; with individual characters, such as stem length and the pod form, the ratio was almost precisely 1:3, as in Pisum.

As limited as the result of this experiment may be for determining the numerical ratios in which the different forms occurred, it does, on the other hand, offer a case of a *curious transformation of colour* in the flowers and seeds of the hybrids. As is known to occur in Pisum, the characters of flower colour and seed colour appear unchanged in the first and subsequent generations, and the progeny of hybrids carry exclusively one or the other of the two original parental characters. Such behaviour is not the case in this experiment. The white flowers and seed colour of Ph. nanus, however, did appear the same in one rather fertile specimen in the first generation, but the other 30 plants developed flower colours that represent different gradations of purple-red to pale violet. The colour of the seed coat was no less different from that of the flower. No plant could be considered perfectly fertile; some set no fruit at all, and with others the pods developed only from the last flowers and never ripened. Well-formed seeds were harvested from only 15 plants. The greatest tendency toward sterility was shown in the forms with predominantly red flowers, in which only four ripe seeds were produced from 16 plants. Three of them had seed markings resembling Ph. multiflorus, but with a more or less pale background colour, and the fourth plant produced only a single seed with a plain brown colour. The forms with prepotently violet-coloured flowers had dark-brown, black-brown, and completely black seeds.

The experiment was continued over two additional generations under similarly unfavourable conditions, as even among the progeny of rather fertile plants once again some were mostly less fertile or completely sterile. Flower and seed colours other than the ones noted did not appear. The forms that produced one or more of the recessive characters in the first generation remained constant for those characters without exception. Of those plants that acquired violet flowers and brown or black seeds, some displayed no change in flower and seed colour in the next generations; but the majority, in addition to completely similar progeny, produced some with white flowers and seed coats. The red-flowered plants remained so infertile that nothing in particular can be said about their further development.

Notwithstanding the many difficulties these observations had to confront, this experiment at least shows that the development of hybrids follows the same law as in Pisum in relation to those characters corresponding to the form of the plant. With respect to the colour characters, however, it seems difficult to find sufficient accordance. Laying aside the fact that a whole array of colours arises from the union of a white a and purple-red colour, from purple to pale violet and white, it is a striking circumstance that of 31 plants that flowered, only one produced the recessive character of white colour, whereas with Pisum such is the case for every fourth plant on average.

But even these enigmatic phenomena might probably be explained according to the laws that are valid for Pisum if one could assume that the flower and seed colour of Ph. multiflorus are a complex of two or more completely independent colours that individually behave like other constant characters of a plant. If flower colour ***A*** were composed of the independent characters ***A*1** + ***A*2** + **...**, that create the total impression of purple-red colour, then through fertilisation with the differing character of white colour ***a*** the hybrid combinations ***A*1*a*** + ***A*2*a*** + **...** would be formed, and similar behaviour would be expected with the corresponding colour of the seed coat. According to the assumption stated above, each of these hybrid colour combinations would be self-sufficient and would thus develop completely independently from the others. One can easily see, then, by combining the individual developmental series, a complete colour series must arise. If, for example, ***A* = *A*_1_** + ***A*_2_**, then the hybrids ***A*_1_*a*** and ***A*_2_*a*** would correspond to the developmental series

$$A_{1}+2A_{1}a+a$$

$$A_{2}+2A_{2}a+a.$$

The members of these series can occur in nine different combinations and each of them represents the designation for another colour:

**Table:** 

**1**	***A*_1_*A*_2_**	**2**	***A*_1_*a A*_2_**	**1**	***A*_2_*a***
**2**	***A*_1_*A*_2_*a***	**4**	***A*_1_*a A*_2_*a***	**2**	***A*_2_*a a***
**1**	***A*_1_*a***	**2**	***A*_1_*a a***	**1**	***a a***

The numbers assumed for the individual combinations simultaneously indicate how many plants with the corresponding colour belong to the series. Since that sum equals 16, all colours, on average, are distributed to each of 16 plants, although, as the series itself shows, in unequal proportions.

If the development of colours actually took place in this manner, the case noted above could be explained—that white flowers and seed colour occurred only once among 31 plants of the first generation. This colour is included only once in the series and could thus, on average, develop once for each 16 plants and, with three colour characters, only once for each 64 plants.

It must not be forgotten, however, that the explanation proposed here is based only on a mere supposition that has no other support than the very imperfect result of the experiment just discussed. It would, of course, be a worthwhile labour to follow the development of colour in hybrids with similar experiments, since it is probable that in this way we would come to understand the extraordinary multitude of *colours in our ornamental flowers*.

At this point, little more is known with certainty other than flower colour in most ornamental plants is an extremely variable character. The opinion has often been expressed that the stability of a species has been disrupted to a high degree or utterly broken through cultivation. There is a common inclination to refer to the development of cultivated forms as proceeding without rules and by chance; the colour of ornamental plants is generally cited as a pattern of instability. It is not apparent, however, why the mere placement in garden soil should result in such a drastic and persistent revolution in the plant organism. No one will seriously assert that the development of plants in a natural landscape is governed by different laws than in a garden bed. Here, just as there, typical variations must appear if the conditions of life are changed for a species, and it has the ability to adapt to the new conditions. It is freely admitted, through cultivation the production of new varieties is favoured, and by the hand of man many a variation is preserved that would have failed in the wild state, but nothing gives us the right to assume that the tendency for new varieties to form is so extremely augmented that species soon lose all stability and that their offspring break up into an infinite array of highly variable forms. If the change in the conditions of vegetation were the sole cause of variability, then one would be justified in expecting that those domesticated plants cultivated under almost the same conditions for centuries would have acquired stability. As is well known, this is not the case, for especially among them not only the most different but also the most variable forms are found. Only the Leguminosae, like Pisum, Phaseolus, Lens, whose organs of fructification are protected by a keel, constitute an appreciable exception. Even for these, numerous varieties have arisen during cultivation for more than 1000 years under the most diversified conditions; however, under the same permanent conditions of life, they retain stability similar to that of species growing in the wild.

It remains more than probable that there is a factor in action for the variability of cultivated plants, which hitherto has received little attention. Different experiences urge us to the view that our ornamental plants, with few exceptions, are *members of different hybrid series* whose legitimate further development is modified and delayed through numerous intercrosses. The circumstance must not be overlooked that cultivated plants usually are raised in larger numbers next to one another, which affords the most favourable opportunity for reciprocal fertilisation between the existing varieties and between species themselves. The probability of this view is corroborated by the fact that among the great host of variable forms, individuals are always found that remain constant in the one or the other character if every foreign influence is carefully prevented. These forms develop precisely the same as certain members of the complex hybrid series. Even with the most sensitive of all characters, that of colour, it cannot escape attentive observation that with individual forms the tendency toward variability occurs in very different degrees. Among plants that descend from *one* spontaneous fertilisation, there are often those whose progeny break up widely in the nature and arrangement of colours, whereas others produce forms with less distinction, and when a larger number individuals is examined, some are found that transmit flower colour unchanged to their progeny. The cultivated species of Dianthus give a demonstrative model. A white-flowering specimen of Dianthus Caryophyllus, which itself derives from a white-flowered variety, was isolated in a glasshouse during the flowering period; the numerous seeds acquired from it produced plants with absolutely the same white flower colour. A similar result was produced with a red-tinged-with-violet race crossed with a white-and-red-striped one. Many others, however, that were protected in the same manner produced progeny with more or less different colours and markings.

Whoever surveys the colours in ornamental plants that arise from similar fertilisations cannot easily avoid the conviction that here too development takes place according to a particular law that possibly can be expressed as the *combination of several independent colour characters*.

## Concluding Remarks

It may not be without interest to compare the observations made herein on Pisum with the results of successful research by the two authorities in this area, Kölreuter and Gärtner. According to their similar views, hybrids either keep, in external appearance, the form that is intermediate between the original parents or approach nearer to the type of the one or the other, sometimes hardly distinguishable from it. Generally, if fertilisation is effected through self-pollination, the seeds produced are of different forms that are distinct from the normal type. As a rule, the majority of the individuals from one fertilisation retain the hybrid form, whereas a few others become more similar to the seed plant and one or another individual appears to be nearer to the pollen plant. However, this does not apply to all hybrids without exception. For some individuals, the progeny more closely approach in part one, and in part the other, parental plant, or they all tend more to one side or the other; some, however, *remain perfectly similar to the hybrid* and continue unchanged. The hybrids of varieties behave like the hybrids of species, but they possess even greater variability in form and a more pronounced tendency to revert to the original parental forms.

In relation to the *attributes* of the hybrids and their resulting regular *development*, the agreement with observations made in Pisum is unmistakable. It is another matter for those cases mentioned as exceptions. Gärtner himself admits that the accurate determination of whether a form is more similar to one or the other of the two parents is often extremely difficult as it depends greatly on the subjective view of the observer. There could, however, be another circumstance that may have contributed to fluctuating and uncertain results in spite of the most careful observation and comparison. Plants that, for the most part, are considered as good species and that are different in a larger number of characters were used for the experiments. When dealing in general with cases of greater or lesser similarity, in addition to those characters that are clearly apparent, those that often are difficult to conceive in words must also be taken into account because, as every plant connoisseur knows, they are nevertheless sufficient to give the forms a strange appearance. Supposing that the development of hybrids takes place according to the laws applicable to Pisum, then the series of each single experiment must include very many forms, since it is well known that the number of terms increases by powers of 3 relative to the number of differing characters. With a relatively small number of experimental plants, then, the result could be only approximately clear and in individual cases could deviate not insignificantly. If, for example, the two original parents differed in seven characters and if 100–200 plants were raised from the seeds of their hybrids to assess the degree of relatedness among the progeny, we can easily see how uncertain the judgment must become, since for seven different characters, the developmental series consists of 2187 differing forms that include 16,384 individuals. One or another relationship could be overrepresented, depending on which forms come by chance in larger numbers into the hands of the observer.

Further, if *dominant* characters, that are simultaneously transmitted completely or nearly unchanged to the hybrid, appear among those that differ, then of the two original parents, the one that possesses the larger number of dominant characters will be more apparent among the members of the developmental series. In the experiments with Pisum alluded to earlier for three differing characters, the dominant characters all belonged to the seed plant. Although the members of the series in their internal nature tend toward both original parental plants equally, in this experiment the seed plant type attained such great predominance that of every 64 plants of the first generation, 54 of them completely resembled it or differed from it in only one character. Under the circumstances, one sees how risky it can be to draw inferences about the internal relatedness of hybrids from external similarities.

Gärtner mentions that in those cases where development was regular, it was not the two original parents that were themselves preserved among the progeny of the hybrids but only single individuals closely related to them. With very extensive developmental series it could not, in fact, transpire otherwise. For seven differing characters, for example, among more than 16,000 progeny of the hybrids, the two original parent forms appear only once each. Consequently, it is not readily probable that the two would be produced among a small number of experimental plants; with some probability, however, one may count on the appearance of individual forms that are close to one of them in the series.

We encounter *an essential difference* with those hybrids that remain constant in their progeny and propagate in the same way as the pure species. According to Gärtner, among these are the *distinctly fertile* hybrids*:* Aquilegia atropurpurea-canadensis, Lavatera pseudolbia-thuringiaca, Geum urbano-rivale, and some Dianthus hybrids and, according to Wichura, hybrids of willow species. This circumstance is especially important for the evolutionary history of plants because constant hybrids acquire the status of *new species*. The truth of this fact has been authenticated by the most preeminent observers and cannot be doubted. Gärtner had the opportunity to follow Dianthus armeria-deltoides through the 10th generation, as it regularly propagated itself in the garden.

With Pisum, experiments showed that hybrids form *different* germ and pollen cells and that herein lies the reason for the variability of their progeny. Likewise, with other hybrids whose progeny behave similarly, we may assume the same cause; however, for those that remain constant, the assumption seems admissible that their fertilising cells are all the same and are identical to the foundational cell of the hybrid. According to the view of famous physiologists, in phanerogams, for the purpose of reproduction, one germ cell and one pollen cell unite into a single cell^2^ that is able to develop into an independent organism through the uptake of matter and the formation of new cells. This development takes place according to a constant law that is founded in the material nature and arrangement of the elements, which succeeds in a viable union in the cell. If the reproductive cells are the same and if they accord to the foundational cell of the mother plant, then the development of the new individual will be governed by the same law that applies to the mother plant. If there is a successful union of a germ cell with a *dissimilar* pollen cell, we must assume that between the elements of both cells that determine their reciprocal differences, there is some sort of counterbalance. The intervening cell that arises becomes the foundation of the hybrid organism whose development necessarily follows another law than for the two original parents. If the balance is assumed to be complete in the sense that the hybrid embryo is formed from similar cells in which the differences *are completely and permanently connected*, then it can be further concluded that the hybrid, like every other autonomous plant species, will remain constant in its progeny. The reproductive cells that are formed in the ovaries and the anthers are the same and are identical to the underlying intervening cell.

In relation to those hybrids whose progeny are *variable*, one might perhaps assume that there is an intervention between the differing elements of the germ and pollen cells so that the formation of a cell as the foundation of the hybrid becomes possible; however, the counterbalance of opposing elements is only temporary and does not extend beyond the life of the hybrid plant. Because no changes are perceptible in the general appearance of the plant throughout the vegetative period, we must further infer that the differing elements succeed in emerging from their compulsory association only during development of the reproductive cells. In the formation of these cells, all existing elements act in a completely free and uniform arrangement in which only the differing ones reciprocally segregate themselves. In this manner the production of as many germ and pollen cells would be allowed as there are combinations of formative elements.

This attempted ascription of the essential distinction of either a *permanent or a temporary association* of the differing cell elements in the development of the hybrids can, of course, be of value only as a hypothesis for which a wide scope of interpretation is possible given the dearth of reliable data. Some justification for the stated view lies in the evidence given for Pisum that the behaviour of each pair of differing characters in hybrid union is independent of the other differences between the two original plants and, further, that the hybrid produces as many types of germ and pollen cells as there are possible constant combination forms. The distinctive characters of two plants can ultimately rest only on differences in the nature and grouping of the elements that are present in their foundational cells in living interaction.

The validity of the set of laws suggested for Pisum requires additional confirmation and thus a repetition of at least the more important experiments would be desirable, for instance the one concerning the nature of the hybrid fertilising cells. An individual observer can easily miss a difference that, even if it at first seems unimportant, can increase in importance in such a way that it may not be neglected for the total result. Whether variable hybrids of other plant species reveal completely identical behaviour must also be determined through experiments; although one might well suppose that for important points a fundamental difference cannot occur since the *unity* of the evolutionary plan of organic life is beyond question.

In conclusion, special mention is deserved for the experiments carried out by Kölreuter, Gärtner, and others on the *transformation of one species into another through artificial fertilisation*. Special importance was set on these experiments; Gärtner counts them among the “most difficult in the production of hybrids.”

For one species ***A*** to be converted into another ***B***, both were united through fertilisation and the hybrids produced were again fertilised with the pollen of ***B***; then the form was selected from the different offspring that was nearest to the species ***B*** and repeatedly fertilised with it and so on until one finally achieved a form that closely resembled ***B*** and remained constant in its progeny. Thus, the species ***A*** was transformed into the other species ***B***. Gärtner himself conducted 30 such experiments with plants from the genera Aquilegia, Dianthus, Geum, Lavatera, Lychnis, Malva, Nicotiana, and Oenothera. The duration for the transformation was not the same for all species. While three fertilisations were sufficient for some, this had to be repeated five to six times with others; also, for these same species fluctuations were observed in different experiments. Gärtner attributes this difference to the circumstance that “the typical vigour with which a species acts in reproduction for changing and modifying the maternal type is very different with different plants, and that as a consequence the time periods within which and the number of generations through which one species is transformed into the other also must be different, so the transformation of some species is achieved through more, and of others through fewer, generations.” Furthermore, the same observer noticed “that it also depends on the transformation processes which type and which individual is chosen for further transformation.”

If one could assume that the development of the forms in these experiments took place in a manner similar to that in Pisum, then the whole transformation process could be explained rather simply. The hybrid forms as many kinds of germ cells as admissible given the constant combinations of its aggregated characters, and one of these is always the same as the fertilising pollen cells. Thus it is possible in all such experiments that as early as the second fertilisation a constant form resembling the pollen plant is acquired. Whether this is actually produced, however, depends in each individual case on the number of experimental plants as well as on the number of the differing characters that are united through fertilisation. Let us assume, for example, that the particular plants for the experiment were different in three characters and the species ***ABC*** was to be transformed into the other ***abc*** through repeated fertilisation with the pollen of that species. The hybrid arising from the first fertilisation forms eight different kinds of germ cells, namely

ABC,ABc,AbC,aBC,Abc,aBc,abC,abc.

These are again combined with the pollen cells ***abc*** in the second experimental year and one obtains the series

AaBbCc+AaBbc+AabCc+aBbCc+Aabc+aBbc+abCc+abc.

Because the form ***abc*** occurs once in the eight-membered series, it is less probable that it would be absent among the experimental plants, even if they were raised in a smaller number, and the transformation would be completed after just two fertilisations. If by chance it were not produced, then the fertilisation would need to be repeated on one of the nearest related combinations ***Aabc***, ***aBbc***, ***abCc***. It becomes apparent that *the smaller the number of experimental plants and the larger the number of differing characters* in the two original parents, the longer such an experiment would have to be drawn out, and further that a postponement of one, or even of two, generations could easily occur with those same species, as Gärtner observed. The transformation of widely divergent species may well be finished only in the fifth or sixth experimental year because the number of different germ cells that are formed in the hybrid increases with the number of differing characters by powers of 2.

Gärtner found through repeated experiments that the *reciprocal* transformation time for some species is different so that often species ***A*** can be converted into another ***B*** one generation earlier than species ***B*** into the other ***A***. He also derives from that evidence that the view of Kölreuter is not completely valid, according to which “the two natures in the hybrid are in perfect balance.” It seems, however, that Kölreuter does not deserve such a criticism and that, more importantly, Gärtner overlooked an important factor to which he himself draws attention elsewhere, that it is, namely, “dependent on which individual is chosen for further transformation.” Experiments in this regard made with two Pisum species indicate that which species is being transformed into the other can make a great difference for the selection of the most suitable individuals for the purpose of further fertilisation. The two experimental plants differed in five characters, and species ***A*** possessed all dominant and the other species ***B*** all recessive characters. For the reciprocal transformation ***A*** was fertilised with the pollen from ***B*** and conversely ***B*** with that from ***A***, and then the same was repeated with both hybrids in the next year. With the first experiment ***B*/*A*** there were in the third experimental year 87 plants, in fact *in all possible 32 forms*, available for selection of individuals for further fertilisation; for the second experiment ***A*/*B*** 73 plants were produced that in their general appearance were thoroughly *identical to the pollen plant*, but according to their internal nature were necessarily as different as the forms of the other experiment. Calculated selection was then possible only in the first experiment; in the second experiment some plants had to be rejected by mere chance. Of the latter only a portion of the flowers were fertilised with the pollen of ***A***, and the others were left to self-fertilise. As the next year’s cultivation showed, of every 5 plants used for fertilisation for the two experiments, there was in accordance with the pollen plant:

**Table:** 

First Experiment		Second Experiment				
2	plants		----	in	all	characters
3	“		----	“	4	“
	----	2	plants	“	3	“
	----	2	“	“	2	“
	----	1	plant	“	1	character

For the first experiment, the transformation was finished; for the second, which was not continued further, two additional fertilisations would probably have been necessary.

Even though the case does not frequently occur in which the dominant characters belong exclusively to the one or the other original parent plant, it still makes a difference *which* of the two possesses the greater number. If the majority of the dominant characters belong to the pollen plant, then the selection of forms for further fertilisation will give a lower degree of certainty compared to the converse case, resulting in a lengthened time required for transformation, supposing that the experiment is viewed as finished only when a form is produced that not only appears the same as the pollen plant in its form but also likewise remains constant in its progeny.

Through the success of transformation experiments, Gärtner was persuaded to oppose the opinion of those naturalists who dispute the stability of plant species and assume continuous evolution of plant species. He sees in the completed transformation of one species into the other the unambiguous evidence that a species has fixed limits beyond which it cannot change. Although this view cannot be afforded unconditional validity, nonetheless a confirmation deserving notice regarding the supposition made earlier about the variability of cultivated plants is found in the experiments performed by Gärtner.

Among the experimental species were cultivated plants, like Aquilegia atropurpurea and canadensis; Dianthus Caryophyllus, chinensis, and japonicas; and Nicotiana rustica and paniculata, and these too had lost none of their autonomy after four or five hybrid unions.

## Notes on the translation:

- British spelling is used throughout the English translation, consistent with the use of Darwinian phraseology.
- In a few instances we used slightly different English words derived from the same root as a Darwinian word when the derived word improved clarity and translation accuracy, such as “special” instead of “especial,” “original” instead of “aboriginal,” and “objective” instead of “object.”
- We translated Mendel’s repeated use of the phrase *je zwei* and its derivatives as “each pair” because in the context of Mendel’s usage this translation is more accurate than the literal translation “each two.” This choice is consistent with the Druery-Bateson and Sherwood-Stern translations.
- We translated Mendel’s repeated use of the phrase *fremder Pollen* as “pollen from another individual,” which is the Darwinian English for *fremder Pollen* in a passage marked by Mendel. It better conveys Mendel’s intended meaning than the literal translation “foreign pollen.”
- We italicized only those words that Mendel italicized in his handwritten proof for the printer. We retained Mendel’s depiction of genus and species names even when incorrect, italicizing them only when Mendel did (contrary to Sherwood and Stern who italicized and corrected genus and species names).

## Footnotes

^1^One variety has a beautiful brown-red pod colour that transforms into violet and blue at the time of ripening. The experiment with this character was begun only during the past year.

^2^With Pisum it is shown without doubt that there must be a complete union of the elements of both fertilising cells for the formation of the new embryo. How could one otherwise explain that among the progeny of hybrids both original forms reappear in equal number and with all their peculiarities? If the influence of the germ cell on the pollen cell were only external, if it were given only the role of a nurse, then the result of every artificial fertilisation could be only that the developed hybrid was exclusively like the pollen plant or was very similar to it. In no manner have experiments until now confirmed that. Fundamental evidence for the complete union of the contents of both cells lies in the universally confirmed experience that it is unimportant for the form of the hybrid which of the original forms was the seed or the pollen plant.

